# Spatial organization of heterologous metabolic system *in vivo* based on TALE

**DOI:** 10.1038/srep26065

**Published:** 2016-05-17

**Authors:** Ling-yun Zhu, Xin-yuan Qiu, Ling-yun Zhu, Xiao-min Wu, Yuan Zhang, Qian-hui Zhu, Dong-yu Fan, Chu-shu Zhu, Dong-yi Zhang

**Affiliations:** 1 College of Science, National University of Defense Technology, Changsha, Hunan, 410073, People’s Republic of China; 2 College of Aerospace Science and Engineering, National University of Defense Technology, Changsha, Hunan, 410073, People’s Republic of China

## Abstract

For years, prokaryotic hosts have been widely applied in bio-engineering. However, the confined *in vivo* enzyme clustering of heterologous metabolic pathways in these organisms often results in low local concentrations of enzymes and substrates, leading to a low productive efficacy. We developed a new method to accelerate a heterologous metabolic system by integrating a transcription activator-like effector (TALE)-based scaffold system into an *Escherichia coli* chassis. The binding abilities of the TALEs to the artificial DNA scaffold were measured through ChIP-PCR. The effect of the system was determined through a split GFP study and validated through the heterologous production of indole-3-acetic acid (IAA) by incorporating TALE-fused IAA biosynthetic enzymes in *E. coli*. To the best of our knowledge, we are the first to use the TALE system as a scaffold for the spatial organization of bacterial metabolism. This technique might be used to establish multi-enzymatic reaction programs in a prokaryotic chassis for various applications.

For years, prokaryotic cells have been widely applied as host cells in bio-engineering and relevant fields because of their rapid growth and high productivities over eukaryotic cells^1^,^2^. However, the weak multi-enzyme co-localization mechanisms in prokaryotes result in relatively low local concentrations of heterologous enzymes and substrates, rapid diffusion and degradation of key intermediates, undesirable crosstalk with native pathways, and accumulation of toxic metabolites^3^,^4^,^5^,^6^. As such, the mechanisms ultimately exhibit a low productive efficacy, especially when generating products through a complex multi-enzymatic cascade. For years, this problem has limited the applications of prokaryotic cells in bio-engineering.

Hence, multiple methods have been designed to accelerate heterologous metabolic pathways by physically enriching the local concentration of enzymes and substrates in certain areas of the cell. Delebecque *et al.* demonstrated a method to increase the hydrogen production of a heterogeneous hydrogen-producing pathway *in vivo* with rationally designed RNA assemblies^4^. Recently, similar methods have been applied *in vivo* on acyl–ACP reductase and aldehyde deformylating oxygenase^7^. Nevertheless, the limited corresponding ligadin and the fragility of RNA itself challenge the expansibility and stability of such a method, which may constrain its further application. Some studies also attempted to modify DNA and proteins as artificial scaffolds for the same purpose^8^,^9^,^10^. However, traditional DNA scaffolds are reportedly difficult to assemble as a single-stranded loop–stem structure to bind the ligadin *in vivo*^11^,^12^. Meanwhile, the complex structure of scaffold proteins challenges the correct protein folding by prokaryotic biofactories, thereby restricting their usage^13^,^14^,^15^. Therefore, both scaffold methods remained limited to *in vitro* applications.

Novel artificial scaffold methods that are natural, highly stable, expandable, and simple must be established to promote the application of spatial organization of enzymes in various metabolic systems and chassis. Recently developed genome-editing tools [e.g., zinc-finger nucleases (ZFN), transcription activator-like effector nuclease (TALEN), and CRISPER/Cas9] can precisely meet this requirement and provide clues for new scaffold systems because of their locus-specific DNA-binding ability. Accordingly, a recent study has applied the CRISPER–Cas9 technique as a scaffold system that concentrates specific enzymes for metaplastic functions^16^. However, this CRISPER–Cas9 scaffold system is essentially consistent with the RNA scaffold that still relies on RNA hairpins linked with gRNA. Thus, the application of such a method is still limited. Conrado *et al.* reported a zinc-finger-based DNA scaffold system to improve catalytic efficiency, indicating a powerful DNA scaffold tool *in vivo* on the basis of the specific and direct combination between DNA and proteins^17^. However, given its simpler design, higher specificity, and lower toxicity compared with ZFN^18^, the TALEN technique might be more efficient and practical for the clustering of multi-enzymes *in vivo* on the basis of TALE-fused enzymes and their corresponding DNA scaffolds.

TALE is a family III effector in *Xanthomonas* that aids the pathogen in infecting various plant species^19^. Different TALEs share a similar domain structure that enables them to bind the genome of the host cell and act as transcriptional effectors. A certain amount (around 1.5–33.5) of tandem transcription activator-like [TAL] repeats, each able to recognize a specific DNA base pair, was determined in the central DNA-binding domain of TALEs^20^,^21^. Each TAL repeat contains 33–35 highly conserved amino acids, among which the residues at positions 12 and 13 (also known as repeat variable di-residues) confer DNA specificity. This structural characteristic allows the TALE to be utilized in protein-engineering applications. TALENs are created by physically fusing the TALE with the cleavage domain of FokI nucleases. These nucleases are extensively applied in prokaryotic and eukaryotic cells. Other methods for engineering TALEs as transcriptional effectors, chromatin regulators, imagers, and separators have also been reported^22^,^23^,^24^,^25^.

In this study, we generate a new method to increase the production of a heterogeneous metabolic system in prokaryotic cells on the basis of the DNA-binding characteristic of TALEs. This artificial TALE–DNA scaffold system can efficiently gather TALE-fused proteins/enzymes around the DNA scaffolds and enrich local enzyme concentrations. The exact function of this method in accelerating exogenous metabolic reactions was tested by a biosynthesis system of indole-3-acetic acid (IAA). IAA is a plant hormone that regulates various developmental and physiological processes^26^ and increases plant protection from external stress^27^ via the spatial organization of two enzymes, namely, tryptophan-2-mono-oxygenase (IAAM) and indole-3-acetimide hydrolase (IAAH). To the best of our knowledge, this study provides a novel method to hasten the heterologous metabolic system in prokaryotic cells. The first introduction of TALEs into artificial *in vivo* scaffold systems can enrich the application of the TALE technique and provide broad insights into the construction of new *in vivo* multi-enzymatic accelerators with high compatibility and extensibility.

## Results

### Evaluation of the binding ability of TALE-GFP1/2 to the plasmid DNA scaffold

**Figure 1:**
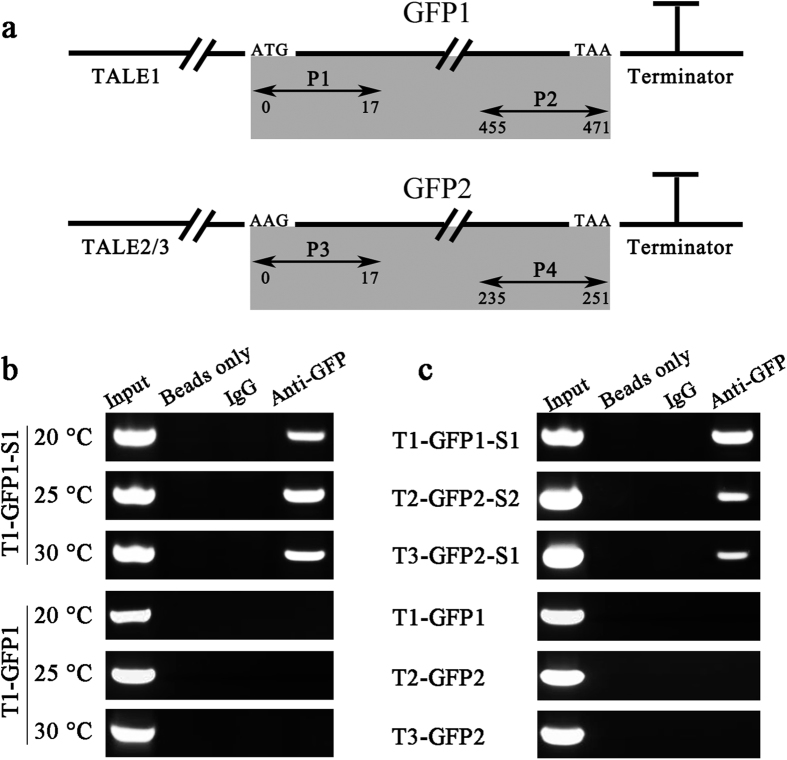
ChIP-PCR assay of TALE–GFP binding to the scaffold in *E. coli*. (**a**) Schematic of the primers and the plasmid regions tested in ChIP assays. P1/P2 was designed for the TALE1–GFP1 ChIP assay, and P3/P4 was used for the TALE2–GFP2 and TALE3–GFP2 ChIP assays. (**b**) Exploration of the optimal temperature for *E. coli* culture upon IPTG induction for the ChIP-PCR assay. (**c**) Determination of the binding abilities of TALE1–GFP1, TALE2–GFP2, and TALE3–GFP2 to their corresponding DNA scaffolds. The groups transformed with their corresponding no-scaffold plasmids were set as controls. Input indicates an aliquot of total DNA. Antibodies used for immunoprecipitation are indicated above the lanes. The cDNA sequence of 16S rRNA was amplified as the standard.

ChIP-PCR analysis was conducted to evaluate whether the TALE–GFP1/2 fusion protein can effectively target the binding motifs (BMs) on the plasmid DNA scaffold. To perform the assay, the synthetic biological parts of the promoter Plac, the ribosome binding site (RBS), TALE1/2/3, split GFP1/2, the terminator, and the corresponding scaffold1/2 were assembled in order by using the BioBrick™ standard assembly method and then inserted into the plasmid backbone of pSB1C3. The plasmids (Supplementary Fig. S1a online) pT1-GFP1-S1, pT2-GFP2-S2, pT3-GFP2-S1, pT1-GFP1 (no-scaffold control), pT2-GFP2 (no-scaffold control), and pT3-GFP2 (no-scaffold control) were verified by sequencing and then transformed into the *Escherichia coli* chassis. The primers used for ChIP-PCR were forward P1 and reverse P2 for GFP1 amplification to detect TALE1–GFP1 binding and forward P3 and reverse P4 for GFP2 amplification to detect TALE2/3–GFP2 binding (Fig. 1a). To optimize the culture temperature during isopropylthio-β-galactoside (IPTG) induction, the TALE1–GFP1–Scaffold1 system was used as a representative for the ChIP-PCR assay. All of the culture temperatures (20 °C, 25 °C, and 30 °C) were suitable for TALE–GFP expression and binding to corresponding scaffolds, among which the 25 °C group showed the strongest binding signal. These results may be attributed to the assumption that a cultivation temperature of 25 °C is relatively appropriate for exogenous protein folding and scaffold stabilization in *E. coli*. Thus, we selected 25 °C as the culture temperature for subsequent experiments (Fig. 1b). As shown in Fig. 1c, 471 and 251 bp DNA fragments were amplified from the precipitates of the TALE1–GFP1–Scaffold1 and TALE2–GFP2–Scaffold2/TALE3–GFP2–Scaffold1 groups, respectively, by using anti-GFP antibody. By contrast, the negative control immunoprecipitations that did not use antibodies (beads only) or adopted the normal rabbit IgG showed no amplification signal. Meanwhile, the no-scaffold control groups did not display any amplification signal. The amplified fragments were confirmed by sequencing. These results indicate that TALE–GFP1/2 can specifically bind to the corresponding plasmid DNA BMs *in vivo*.

### Split GFP assay for determining the effectiveness of the TALE–DNA scaffold system

**Figure 2:**
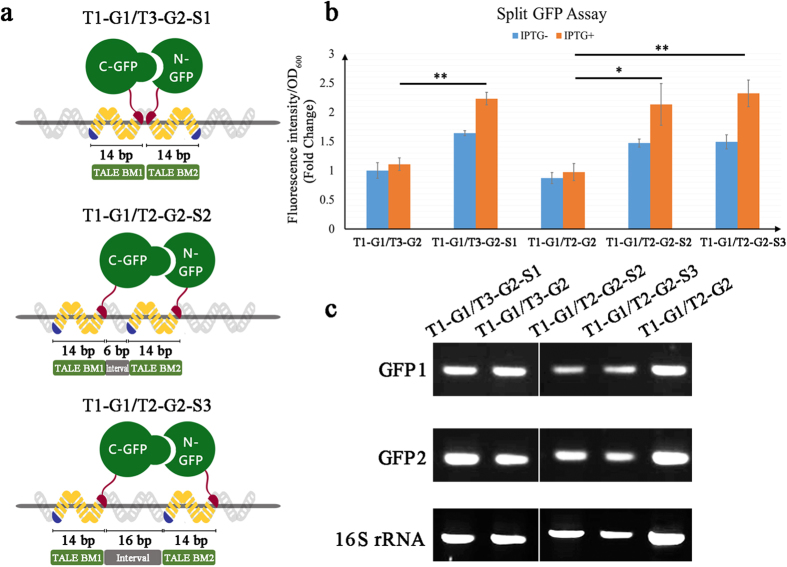
Evaluation of the TALE–DNA scaffold system by split GFP assay. (**a**) Schematic of the manner of integration of split GFP to the TALE–DNA scaffold system and their hypothetical binding patterns to DNA BMs. The designed DNA BMs are shown at the top left of each panel. These patterns include the “head-to-head” pattern (upper panel) and the “BM1-interval-BM2-interval” pattern with 6 bp (middle panel) and 16 bp intervals (lower panel). (**b**) The green fluorescence (Ex: 488 nm; Em: 538 nm) of split GFP was examined after overnight culture of *E. coli* with or without IPTG induction. Relative fluorescence intensity was calculated with normalization of the OD_600_ value. The relative fluorescence intensity of the TALE1–GFP1/TALE3–GFP2 control group was set arbitrarily at 1.0, and the levels of the other groups were adjusted correspondingly. This experiment was run in three parallel reactions, and the data represent results obtained from at least three independent experiments. *p < 0.05, **p < 0.01. (**c**) Semi-qRT-PCR analysis of GFP1 and GFP2 expression in different TALE–GFP–scaffold groups. The cDNA sequence of 16S rRNA was amplified as the standard.

The effect of the TALE–DNA scaffold system on the *in vivo* clustering of functional proteins fused with orthogonal interaction domains was examined using the split GFP assay. To perform this assay, the synthetic biological parts of Plac, RBS, TALE1, GFP1, TALE2/3, GFP2, the terminator, and the corresponding scaffold1/2/3 were assembled in order using the BioBrick™ standard assembly method and then inserted into the plasmid backbone of pSB1C3. The plasmids pT1-G1/T3-G2-S1, pT1-G1/T2-G2-S2, pT1-G1/T2-G2-S3 (designed as shown in Fig. 2a), pT1-G1/T3-G2 (without scaffold1 control), and pT1-G1/T2-G2 (without scaffold2/3 control) (Supplementary Fig. S1b online) were constructed and then verified by sequencing. *E. coli* BL21 (DE3) transformed with such plasmids were cultured and induced by IPTG overnight. The groups without IPTG supplementation were designated as uninduced controls. Subsequently, bacterial samples were harvested, and corresponding fluorescence intensities (abbreviated as FI, Ex: 488 nm; Em: 538 nm) were determined by the Fluoroskan Ascent FL (Thermo Scientific). As shown in Fig. 2b, the FI/OD_600_ value in the TALE1–GFP1/TALE3–GFP2–Scaffold1 group was significantly higher than that in the no-scaffold1 control group upon IPTG induction (*p* *=* 0.0052). In parallel, the FI/OD_600_ values in the TALE1–GFP1/TALE2–GFP2–Scaffold2 and TALE1–GFP1/TALE2–GFP2–Scaffold3 groups were significantly higher than that in the no-scaffold2/3 control group upon IPTG induction (*p* *=* 0.0100 and *p* *=* 0.0027, respectively). Both semi-quantitative reverse-transcription (semi-qRT-PCR) and real-time quantitative RT-PCR (qRT-PCR) analyses showed that there are no statistical significances in expression of GFP1 (for scaffold1 group *p* = 0.2928, for scaffold2 group *p* = 0.6690, for scaffold3 group *p* = 0.5502) and GFP2 (for scaffold1 group *p* = 0.4745, for scaffold2 group *p* = 0.7877, for scaffold3 group *p* = 0.6177) between scaffold groups and no-scaffold control groups. This result indicated that the increase in green FI in the scaffold system was not caused by the expression variation of GFP1 or GFP2 (Fig. 2c, Fig. S2a,S2b). These findings suggest that the TALE–DNA scaffold system is an efficient device for the clustering and ordering of different proteins fused with TALE proteins.

### Role of the TALE–DNA scaffold system in IAA production

**Figure 3:**
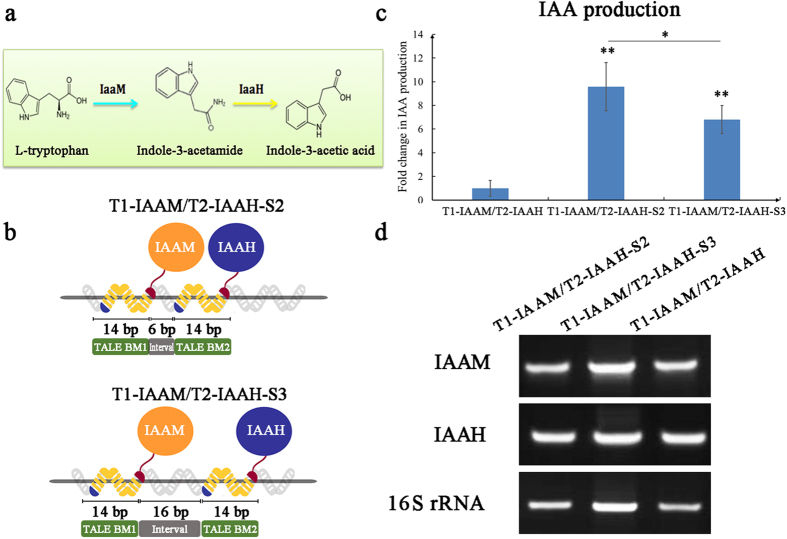
Increase in IAA production by incorporating IAAM and IAAH into the TALE–DNA scaffold system. (**a**) The IAA production pathway converts L-Trp into IAA through IAAM and IAAH. (**b**) Schematic of the manner of integration of IAAM and IAAH into the TALE–DNA scaffold system and their hypothetical binding patterns to the DNA motifs. The designed DNA BMs are shown at the top left of each panel. These patterns include the “BM1-interval-BM2-interval” pattern with 6 bp (middle panel) and 16 bp intervals (lower panel). (**c**) IAA production was determined by sandwich ELISA after overnight culture of *E. coli* with IPTG induction. After TMB staining and reaction termination, the color was measured spectrophotometrically at 450 nm wavelength. IAA concentrations in the samples were then determined by comparing their OD_450_ to standard curves. The relative IAA concentration of the TALE1–IAAM/TALE2–IAAH no-scaffold control group was set arbitrarily at 1.0, and the levels of the other groups were adjusted correspondingly. Data represent the results obtained from at least three independent experiments. *p < 0.05, **p < 0.01. (**d**) Semi-qRT-PCR analysis of IAAM and IAAH expression in different TALE–IAA–scaffold groups. The cDNA sequence of 16S rRNA was amplified as the standard.

To investigate the function of the TALE–DNA scaffold system in heterologous metabolic pathways, we adopted a prototype of the IAA synthetic pathway by fusing the IAAM and IAAH enzymes to two TALEs (TALE1 and TALE2, Fig. 3a,b). After performing a construction process similar to that described above, the plasmids pT1-IAAM/T2-IAAH-S2 and pT1-IAAM/T2-IAAH-S3 (Supplementary Fig. S1c online) were verified by sequencing and then employed to transform *E. coli* BL21 (DE3), which were cultured and induced with IPTG overnight. The pT1-IAAM/T2-IAAH transformation group was applied as the no-scaffold control. Sandwich enzyme-linked immunosorbent analysis (ELISA) revealed that the IAA productions in the T1-IAAM/T2-IAAH-S2 (9.457 μmol/L) and T1-IAAM/T2-IAAH-S3 (6.715 μmol/L) groups were approximately 9.6-fold (*p* = 0.0086) and 6.8-fold (*p* = 0.0028) higher than that in the no-scaffold T1-IAAM/T2-IAAH control group (0.986 μmol/L), respectively (Fig. 3c). Notably, the Scaffold3 group with larger adjacent BM intervals than the Scaffold2 group showed a lower increasing yield efficiency (*p* = 0.0305), indicating a distance-dependent pattern in the TALE–DNA scaffold system. Both semi-qRT-PCR and qRT-PCR analyses showed that there are no statistical significances in expression of IAAM (for scaffold2 group p = 0.4196, for scaffold3 group p = 0.6543) and IAAH (for scaffold2 group p = 0.1079, for scaffold3 group p = 0.7730) between scaffold groups and no-scaffold control group. This result indicated that the increased IAA production in the scaffold system was not due to the expression variation of IAAM and IAAH (Fig. 3d and Fig. S2c). On the basis of these results, the TALE–DNA scaffold system was demonstrated to increase IAA production effectively through an IAAM–IAAH metabolic pathway. Thus, the TALE–DNA scaffold system can efficiently accelerate the rates of heterologous metabolic pathways in prokaryotic chassis.

## Discussion

Given their sequence-specific DNA-binding abilities, TALEs have been extensively used for genome editing, transcriptional regulation, epigenetic modification, visualization of genomic regions, and locus-specific ChIP^28^. All of these applications are focused on genomic research. However, whether the locus-specific DNA-binding behavior of TALEs could be utilized in biochemical and bioengineering studies, such as the hastening of heterologous metabolic reactions *in vivo*, remains undetermined. For this purpose, we first constructed expression plasmids containing both the sequences encoding the rationally designed TALE proteins fused with split GFPs or specific enzymes and the corresponding DNA BMs. After transforming into *E. coli*, both the split GFP and IAA production assays demonstrated that the TALE–DNA scaffold system can be efficiently used to cluster and order TALE-fused proteins, as well as accelerate the rates of heterologous metabolic pathways in prokaryotic chassis.

Previous artificial scaffolds for pathway organization have rapidly become an important approach to provide control over pathway fluxes by altering local enzyme stoichiometry. Consequently, the TALE-based DNA scaffold system developed in this study could be a novel approach to achieve this purpose. The TALE-based DNA scaffold system may possess more advantages in several aspects, such as compatibility, stabilization, expandability, and predictability. TALE is a natural component of the bacterial infection system. Therefore, the TALE-based DNA scaffold system may exhibit substantial compatibility with prokaryotic cells. This feature renders this system suitable for *in vivo* integration into prokaryotic biofactories. The TALE-based DNA scaffold system also exhibits greater extensibility as compared with previously developed *in vivo* protein-based and RNA-based scaffolds. *In vivo* protein scaffolds have encountered the problems of limited metazoan signaling interaction proteins and complexity in folding and function. Although the *in vivo* RNA scaffold system can offer greater predictability and may avoid much of the undesirable crosstalk that is prevalent in protein-based scaffolds, this system still showed degradable properties and fewer well-characterized RNA binding domains of high binding affinity. By contrast, each TALE can specifically bind to a DNA sequence of only 10–20 bp in length. Therefore, tandem DNA BMs can recruit a series of TALE-fused enzymes, which may theoretically allow unlimited numbers of enzymes to be spatially organized. Meanwhile, TALE-based genome manipulation has been confirmed to be available for a diverse array of eukaryotic species, such as mouse, zebrafish, *Caenorhabditis elegans*, and *Drosophila*^29^,^30^,^31^,^32^. This advance suggests the potential of the TALE–DNA scaffold system to be used in broader chassis. Thus, aside from other scaffold systems used to accelerate heterologous metabolic pathways, the TALE–DNA scaffold system theoretically possesses a high extensibility both in long multi-enzymatic chains and in numerous host species. However, this supposition remains to be confirmed in future studies.

According to previous reports, the importance of the precise order and spacing of enzymes in the context of pathway enhancement remains debatable^33^. Accordingly, our results indicated that the influence of interval length between adjacent TALE-binding motifs to the TALE system was fused-enzyme/protein type-dependent. For instance, the IAA production system showed a distance-dependent pattern, whereas the split-GFP system did not. These differences may be attributed to the fact that the IAA production system essentially depended on an enzyme-catalytic mechanism, whereas the split-GFP system was essentially influenced by a protein interaction mechanism. As indicated by the scaffold-mediated metabolite micro-domain hypothesis^34^, different scaffold architectures with different parameters, i.e., enzyme distance, may significantly affect the probability of the intermediate’s downstream reaction and the subsequent metabolic flux dynamics in the micro-domain. This hypothesis explains the distance-dependent pattern of IAA production. However, as long as the distance between the two portions of GFP is within a tolerable range for their interaction in the split GFP assay, the effect of subtle distance variation between these parts might be insignificant. Meanwhile, the enzyme-catalytic process might amplify the distance effect, whereas the split GFP assay could not exert such an effect. Nevertheless, the space effect on different metabolic systems requires further investigations based on TALE–DNA scaffolds in future studies to completely elucidate such mechanisms.

To the best of our knowledge, this study is the first to use TALEs in an *in vivo* artificial heterologous metabolic accelerating system in a prokaryotic chassis. Our study demonstrated that locus-specific DNA-binding tools could be further expanded into broader areas, such as biochemical engineering, in addition to traditional genome studies. We anticipate that this novel heterologous metabolic system accelerator would be widely applied both in prokaryotic and eukaryotic biofactories for the bioengineering of various metabolic products, such as biomass, biofuels, and biomaterials, as well as for sewage disposal and waste treatment.

## Methods

### Strains and plasmids

*E. coli* DH5α (Takara) was used for routine subcloning, and *E. coli* BL21 (DE3) was used for protein expression in accordance with the manufacturer’s instructions. The plasmids were constructed on the basis of the BioBrick standard assembly using pSB1C3 as the backbone containing tandem restriction sites for EcoRI, PstI, SpeI, and XbaI digestion. The plasmids of pSB1C3-Plac (BBa_R0010), pSB1C3-IAA biosynthetic genes (BBa_K515100), and pSB1C3-Terminator (BBa_B0015) were supplied by the registry of standard biological parts from the iGEM Foundation. All plasmid manipulations were conducted using standard cloning techniques.

### Design of the DNA scaffold

Two 14 bp TALE BMs (BM1: GGAGGCACCGGTGG and BM2: GATAAACACCTTTC) were designed on the basis of the sequence of an unrelated gene to avoid homology with the *E. coli* genome. Considering the typical *in vivo* B-type DNA structure with helical turns of 10 bp in length, we designed the interval sequences between two 14 bp BMs to be 6 bp or 16 bp long to ensure that the adjacent fusing enzymes are in the same spatial direction. No interval sequence (only a 5′–T for TALE binding) was added to the “head-to-head” pattern to allow the fused enzymes to be as close as possible. The devices exhibiting the “BM1-interval-BM2-interval” pattern were synthesized with repetitions of more than 10 times (11 times) and then integrated into the plasmid (Supplementary Fig. S3 online).

### Construction of TALE-coding plasmid

TALE1, TALE2, and TALE3 were designed on the basis of the DNA scaffold BMs of BM1, BM2, and reverse BM2, respectively. The TALEs were constructed with the Golden Gate TALEN and TAL Effector Kit 2.0 by Addgene (Supplementary Fig. S4 online). The fragments expressing TALE modules were subcloned into a pSB1C3 plasmid flanked by the restriction sites EcoRI and XbaI at the 5′ end and SpeI and PstI at the 3′ end. The linker sequence ACTAGA for the fusion of enzymes with the TALEs was introduced by the isoschizomers of SpeI and XbaI. The primers employed to identify the sequences containing the TALE modules are shown in Supplementary Table S1 online. Plasmid DNA was extracted following the Miniprep protocol (Qiagen) and then sequenced on the MegaBACE 1000 system (GE Healthcare) by using a DYEnamic ET dye terminator cycle sequencing kit (Pharmacia). Full-length cDNA fragments were assembled by a catabolite gene activator protein (CAP 3.0).

### ChIP-PCR assay

For the ChIP-PCR assay, the plasmids pSB1C3-Plac-RBS-TALE1-GFP1-Ter-Scaffold1 (pT1-GFP1-S1), pSB1C3-Plac-RBS-TALE2-GFP2-Ter-Scaffold2 (pT2-GFP2-S2), pSB1C3-Plac-RBS-TALE3-GFP2-Ter-Scaffold1 (pT3-GFP2-S1), pSB1C3-Plac-RBS-TALE1-GFP1-Ter (pT1-GFP1, no-scaffold control), pSB1C3-Plac-RBS-TALE2-GFP2-Ter (pT2-GFP2, no-scaffold control), and pSB1C3-Plac-RBS-TALE3-GFP2-Ter (pT3-GFP2, no-scaffold control) were constructed and transformed into *E. coli* BL21 (DE3) (Supplementary Fig. S1a online). Single colonies harboring the expression plasmids were inoculated into Luria–Bertani medium (100 mL) containing chloramphenicol (35 mg/L). The colonies were then incubated with shaking at 37 °C until an OD_600_ value of 0.6 was reached. Subsequently, IPTG was added to a final concentration of 1 mM and then continually cultured at 20 °C, 25 °C, or 30 °C to determine the optimized induction temperature. Afterward, bacteria were lysed and cross-linked in 1% formaldehyde without ultrasonic treatment because of the small size of the binding plasmid. Immunoprecipitation and DNA purification were performed in accordance with the instructions of the ChIP kit with anti-GFP polyclonal antibody (Abcam). TALE BMs contain highly repeated sequences, and their flanking sequences are homologous to the other parts of the harboring plasmid. Hence, the BMs are difficult to amplify by PCR. However, the binding plasmids have not been ultrasonically treated during ChIP. Thus, the PCR primers used for GFP1 and GFP2 amplifications were employed to detect TALE1–GFP1 and TALE2/3–GFP2 binding, respectively (as shown in Supplementary Table S1 online). The PCR program comprised 30 cycles of treatment at 94 °C for 40 s, 55 °C for 60 s, and 72 °C for 30 s. Finally, 5 μL of PCR products was loaded onto a 1.5% (w/v) agarose gel and visualized by gel staining with 0.1 mg/mL ethidium bromide.

### Split GFP assay

Split GFP was designed and cloned in accordance with previous reports^4^,^35^. In brief, GFP1 and GFP2 fragments were amplified using pEGFP (Clontech) as the template. The fusion genes of TALE1–GFP1 and TALE2–GFP2, as well as the promoter Plac, RBS, terminator, and scaffold sequences, were integrated into pSB1C3 to construct the following plasmids: pSB1C3-Plac-RBS-TALE1-GFP1-RBS-TALE3-GFP2-Ter-Scaffold1 (pT1-G1/T3-G2-S1), pSB1C3-Plac-RBS-TALE1-GFP1-RBS-TALE2-GFP2-Ter-Scaffold2 (pT1-G1/T2-G2-S2), pSB1C3-Plac-RBS-TALE1-GFP1-RBS-TALE2-GFP2-Ter-Scaffold3 (pT1-G1/T2-G2-S3), pSB1C3-Plac-RBS-TALE1-GFP1-RBS-TALE3-GFP2-Ter (pT1-G1/T3-G2, no-scaffold control), and pSB1C3-Plac-RBS-TALE1-GFP1-RBS-TALE2-GFP2-Ter (pT1-G1/T2-G2, no-scaffold control) (Supplementary Fig. S1b online). *E. coli* BL21(DE3) cells were transformed with these plasmids, cultured at 37 °C until the OD_600_ value reached 0.6, and then induced at 25 °C using 1 mM IPTG. The bacteria were harvested, washed, and then coated on a 96-well plate. The fluorescent intensity (Ex: 488 nm; Em: 538 nm) and OD_600_ of each well were measured by Thermo Scientific Fluoroskan Ascent FL. Each sample was measured in three parallel wells, and three independent experiments were performed. For semi-qRT-PCR and qRT-PCR analyses, the total RNA of the IPTG-induced bacteria was isolated using TRIzol reagent (Life Technologies BRL). The RNA isolates were treated with RNase-free DNase I (Qiagen) and then reverse transcribed into first-strand cDNA by using an RNA PCR kit in accordance with the manufacturer’s instructions (PrimeScript™ RT reagent Kit with gDNA Eraser; TaKaRa). The semi-qRT-PCR program and product visualization were conducted as described above. For the qRT-PCR assay, all reactions were performed with a SYBR Premix Ex Taq kit (TaKaRa) in a total volume of 10 μl. PCR program was 94 °C for 2 min, followed by 40 cycles at 94 °C for 20 s, 55 °C for 20 s, and 68 °C for 20 s. Relative gene-expression level was calculated by the 2^−ΔΔCT^ method normalized to 16S rRNA gene. The primers used for the expression of GFP1, GFP2 and 16S rRNA genes are shown in Supplementary Table S1 online.

### ELISA assay for IAA production

For the IAA production assay, the IAAM and IAAH genes were cloned from the plasmid of pSB1C3-IAA biosynthetic genes (BBa_K515100). The plasmids pSB1C3-Plac-RBS-TALE1-IAAM-RBS-TALE2-IAAH-Ter-Scaffold2 (pT1-IAAM/T2-IAAH-S2), pSB1C3-Plac-RBS-TALE1-IAAM-RBS-TALE2-IAAH-Ter-Scaffold3 (pT1-IAAM/T2-IAAH-S3), and pSB1C3-Plac-RBS-TALE1-IAAM-RBS-TALE2-IAAH-Ter (pT1-IAAM/T2-IAAH, no-scaffold control) were constructed and transformed into *E. coli* BL21 (DE3) (Supplementary Fig. S1c online). After transformation, positive colonies were cultured at 37 °C until the OD_600_ value reached 0.6 before induction with 1 mM IPTG for 6 h. Afterward, 2 mL of the bacterial suspension was centrifuged, and the precipitate was lysed and subjected to ELISA. A 96-well plate was coated with capture anti-IAA antibody (Abcam) overnight at 4 °C. The plate was washed three times with PBST and then blocked with 2% bovine serum albumin at 37 °C for 2 h. Subsequently, the samples and standards (2.4, 1.6, 0.8, 0.4, 0.2, and 0 nmol per well) diluted in carbonate buffer (pH 9.6) were incubated for 30 min at 37 °C. After washing and blotting with paper towels, the wells were incubated with detection anti-IAA antibody conjugated with horseradish peroxidase (Abcam) for 30 min at 37 °C. After the plates were washed, color was developed using tetramethylbenzidine (TMB) and then analyzed on a plate reader at 450 nm. The IAA concentration (μmol/L bacterial suspension) of each sample was calculated by the linear function of the IAA standard concentration curve. For semi-qRT-PCR and qRT-PCR analyses, the cDNA of each group was acquired and the PCR programs were conducted in accordance with the above-mentioned methods. The primers used for the expression of IAAM, IAAH, and 16S rRNA genes are shown in Supplementary Table S1 online.

### Statistical analysis

Statistical evaluation of differences between means of the experimental groups was performed using ANOVA and two-tailed t-tests. Both *p* < 0.05 and *p* < 0.01 were considered significant. Data points represented the means of three independent experiments.

## Additional Information

**How to cite this article**: Zhu, L.-y. *et al.* Spatial organization of heterologous metabolic system *in vivo* based on TALE. *Sci. Rep.* **6**, 26065; doi: 10.1038/srep26065 (2016).

## Supplementary Material

Supplementary Information
