# Erratum: Spatial organization of heterologous metabolic system *in vivo* based on TALE

**DOI:** 10.1038/srep27321

**Published:** 2016-06-10

**Authors:** Lv-yun Zhu, Xin-yuan Qiu, Ling-yun Zhu, Xiao-min Wu, Yuan Zhang, Qian-hui Zhu, Dong-yu Fan, Chu-shu Zhu, Dong-yi Zhang

Scientific Reports 6: Article number: 2606510.1038/srep26065; published online: 05172016; updated: 06102016.

The original HTML version of this Article contained typographical errors in the spelling of the author Lv-yun Zhu which was incorrectly given as Ling-yun Zhu.

In addition, Lv-yun Zhu was incorrectly listed as a corresponding author, rather than Ling-yun Zhu.

These errors have now been corrected.

