# Supplementary material for: Spatial organization of heterologous metabolic system in vivo based on TALE
Source: Sci Rep. 2016 May 17;6:26065. doi: 10.1038/srep26065 (PMC4869064; doi:10.1038/srep26065)
Supplement: Supplementary Information [file srep26065-s1.pdf]

Supplementary Information

Spatial organization of heterologous metabolic system *in vivo* based on TALE

Lv-yun Zhu[+, 1], Xin-yuan Qiu[+, 2], Ling-yun Zhu[+, 1,*], Xiao-min Wu[1], Yuan Zhang[2], Qian-hui Zhu[2], Dong-yu Fan[1], Chu-shu Zhu[1], Dong-yi Zhang[1,*]

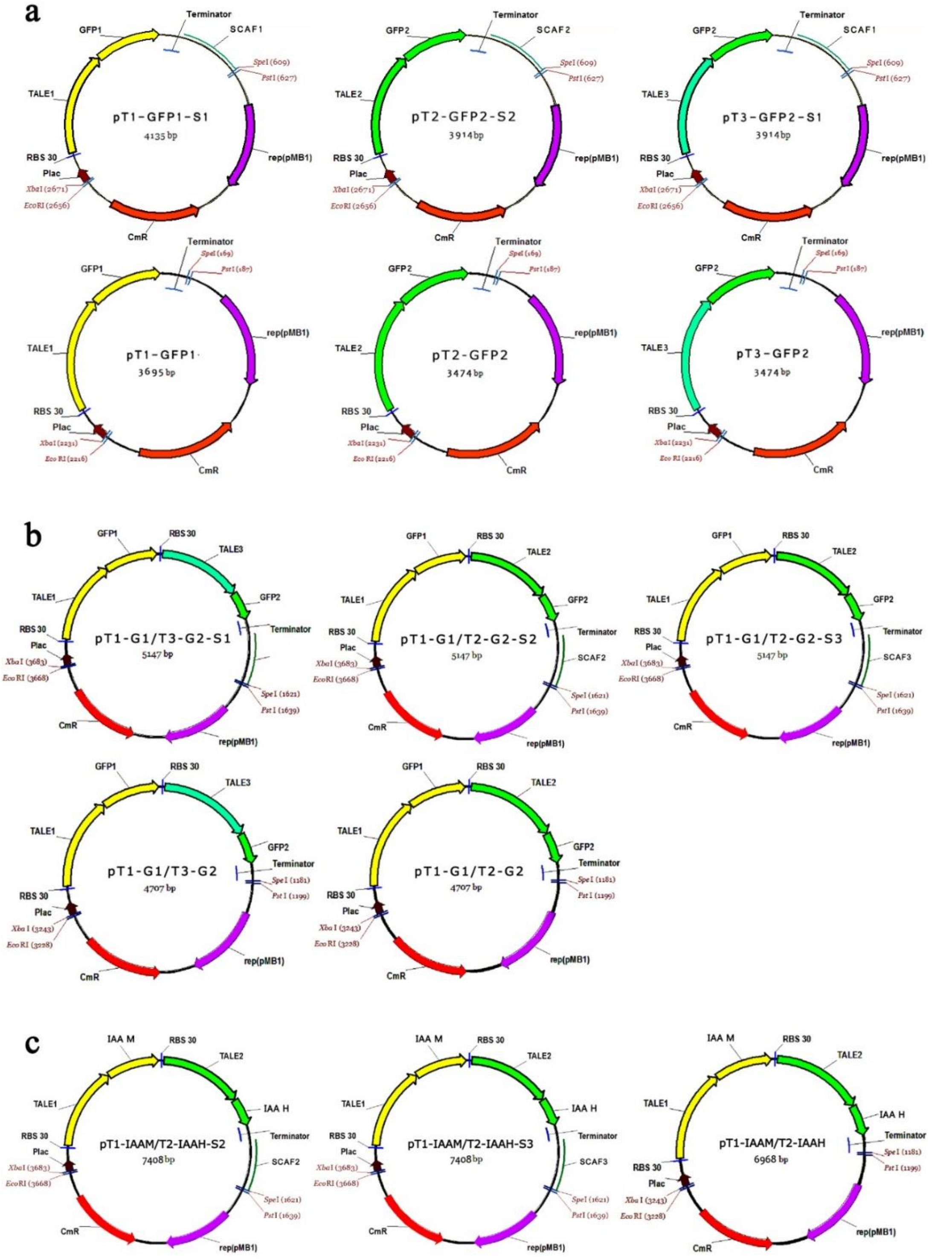

Supplementary Figure S1. Schematic representations of the constructed plasmids. (**a**) Plasmids used for the ChIP-PCR assay. (**b**) Plasmids used for the split-GFP assay. (**c**) Plasmids used for the IAA production assay.

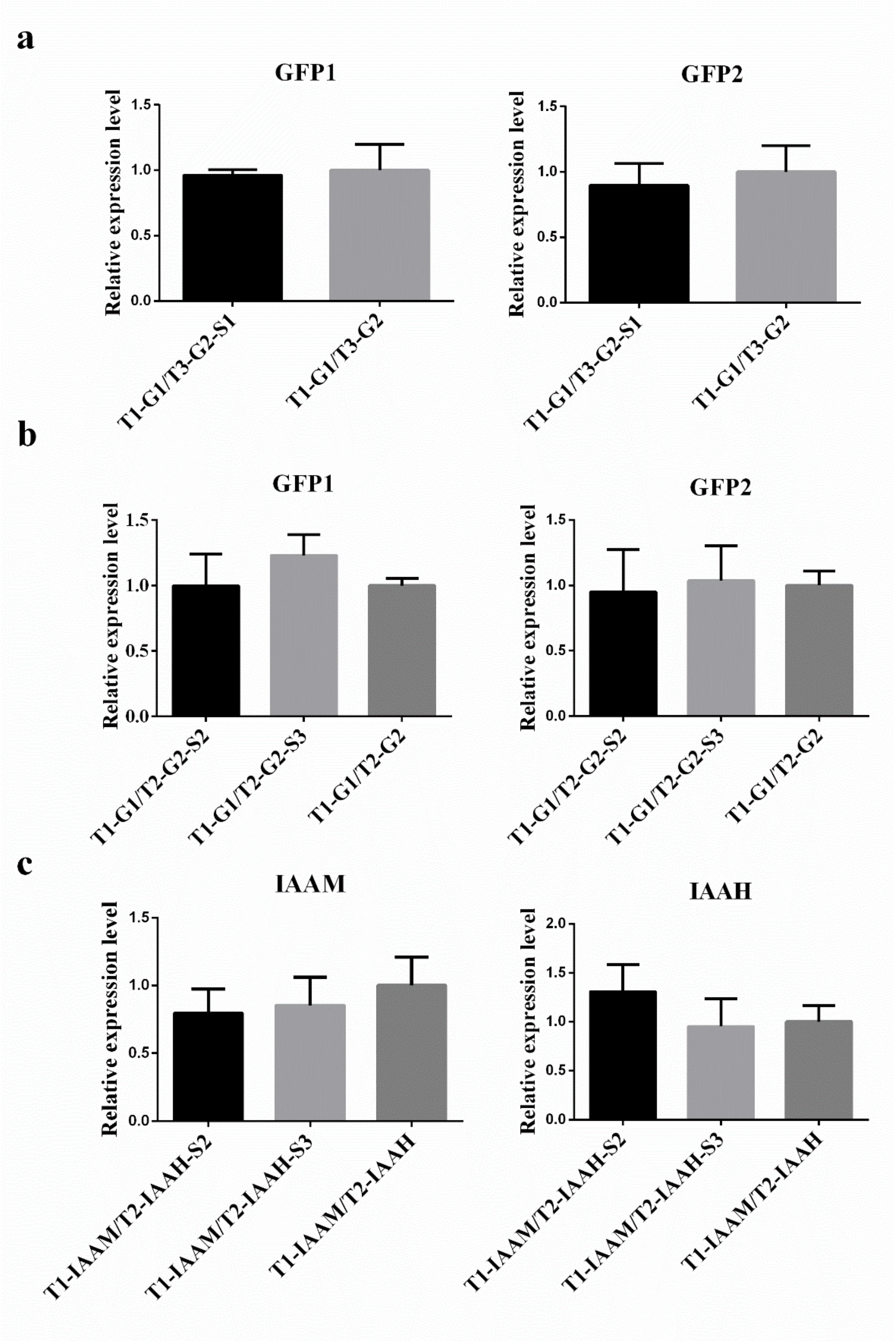

Supplementary Figure S2. qRT-PCR analysis testing the gene expression levels of split GFP and specific enzymes among different scaffold and no-scaffold groups. (**a**) qRT-PCR analysis of GFP1 and GFP2 expression between TALE1-GFP1/TALE3-GFP2-

Scaffold1 and the corresponding no-scaffold groups. (**b**) qRT-PCR analysis of GFP1 and GFP2 expression among TALE1-GFP1/TALE2-GFP2-Scaffold2, TALE1-GFP1/TALE2-GFP2-Scaffold3 and the corresponding no-scaffold groups. (**c**) qRT-PCR analysis of IAAM and IAAH relative expression level among TALE1-IAAM/TALE2-IAAH-Scaffold2, TALE1-IAAM/TALE2-IAAH-Scaffold3 and the corresponding no-scaffold groups. Relative gene expression was calculated using the $2^{-\Delta\Delta CT}$ method, with initial normalization of genes against 16s rRNA gene within each treatment group. The expression levels of each gene in the corresponding no-scaffold control groups were arbitrarily set to 1.0. The relative expression values were averaged from the data in three parallel reactions, and the results were obtained from at least three independent experiments. Error bars represent SE.

a

GTTTCTTC*GAATTC*GCGGCCGCT*TCTAGA*TGGAGGCACCGGTGGT
EcoRI XbaI
GATAAACACCTTTCTGGAGGCACCGGTGGTGATAAACACCTTTCT
GGAGGCACCGGTGGTGATAAACACCTTTCTGGAGGCACCGGTGG
TGATAAACACCTTTCTGGAGGCACCGGTGGTGATAAACACCTTTC
TGGAGGCACCGGTGGTGATAAACACCTTTCTGGAGGCACCGGTG
GTGATAAACACCTTTCTGGAGGCACCGGTGGTGATAAACACCTTT
CTGGAGGCACCGGTGGTGATAAACACCTTTCTGGAGGCACCGGT
GGTGATAAACACCTTTCTGGAGGCACCGGTGGTGATAAACACCTT
TC*ACTAGT*AGC
SpeI

b

GTTTCTTC*GAATTC*GCGGCCGCT*TCTAGA*TGGAGGCACCGGTGGA
EcoRI XbaI
CAGTTGATAAACACCTTTCACAGTTGGAGGCACCGGTGGACAGT
TGATAAACACCTTTCACAGTTGGAGGCACCGGTGGACAGTTGAT
AAACACCTTTCACAGTTGGAGGCACCGGTGGACAGTTGATAAAC
ACCTTTCACAGTTGGAGGCACCGGTGGACAGTTGATAAACACCT
TTCACAGTTGGAGGCACCGGTGGACAGTTGATAAACACCTTTCA
CAGTTGGAGGCACCGGTGGACAGTTGATAAACACCTTTCACAGT
TGGAGGCACCGGTGGACAGTTGATAAACACCTTTCACAGTTGGA
GGCACCGGTGGACAGTTGATAAACACCTTTCACAGTTGGAGGCA
CCGGTGGACAGTTGATAAACACCTTTCACAGTTGGAGGCACCGG
TGGACAGTTGATAAACACCTTTCACAGTT*ACTAGT*AGC
SpeI

c

GTTTCTTC*GAATTC*GCGGCCGCT*TCTAGA*TGGAGGCACCGGTGGA
EcoRI XbaI
CAGTAGCTACAACCTGATAAACACCTTTCACAGTAGCTACAACCT
GGAGGCACCGGTGGACAGTAGCTACAACCTGATAAACACCTTTC
ACAGTAGCTACAACCTGGAGGCACCGGTGGACAGTAGCTACAAC
CTGATAAACACCTTTCACAGTAGCTACAACCTGGAGGCACCGGT
GGACAGTAGCTACAACCTGATAAACACCTTTCACAGTAGCTACAA
CCTGGAGGCACCGGTGGACAGTAGCTACAACCTGATAAACACCT
TTCACAGTAGCTACAACCTGGAGGCACCGGTGGACAGTAGCTAC
AACCTGATAAACACCTTTCACAGTAGCTACAACCTGGAGGCACC
GGTGGACAGTAGCTACAACCTGATAAACACCTTTCACAGTAGCTA
CAACCTGGAGGCACCGGTGGACAGTAGCTACAACCTGATAAACA
CCTTTCACAGTAGCTACAACCTGGAGGCACCGGTGGACAGTAGC
TACAACCTGATAAACACCTTTCACAGTAGCTACAACCTGGAGGCA
CCGGTGGACAGTAGCTACAACCTGATAAACACCTTTCACAGTAGC
TACAACCTGGAGGCACCGGTGGACAGTAGCTACAACCTGATAAA
CACCTTTCACAGTAGCTACAACCT*ACTAGT*AGC
SpeI

Supplementary Figure S3. Sequence of synthesized DNA scaffold. (**a**) Sequence of synthesized DNA scaffold1 without interval (only a 5'–T for TALE binding). The pattern of "head-to-head" is repeated for 11 times. (**b**) Sequence of synthesized DNA scaffold2 with 6 bp interval. The pattern of "BM1-interval-BM2-interval" is repeated for 11 times. (**c**) Sequence of synthesized DNA scaffold3 with 16 bp interval. The pattern of "BM1-interval-BM2-interval" is repeated for 11 times. The BMs are shaded, and the sequences of BM1, interval and BM2 are indicated in blue, red and green, respectively. The restriction sites are marked in italic and underlined.

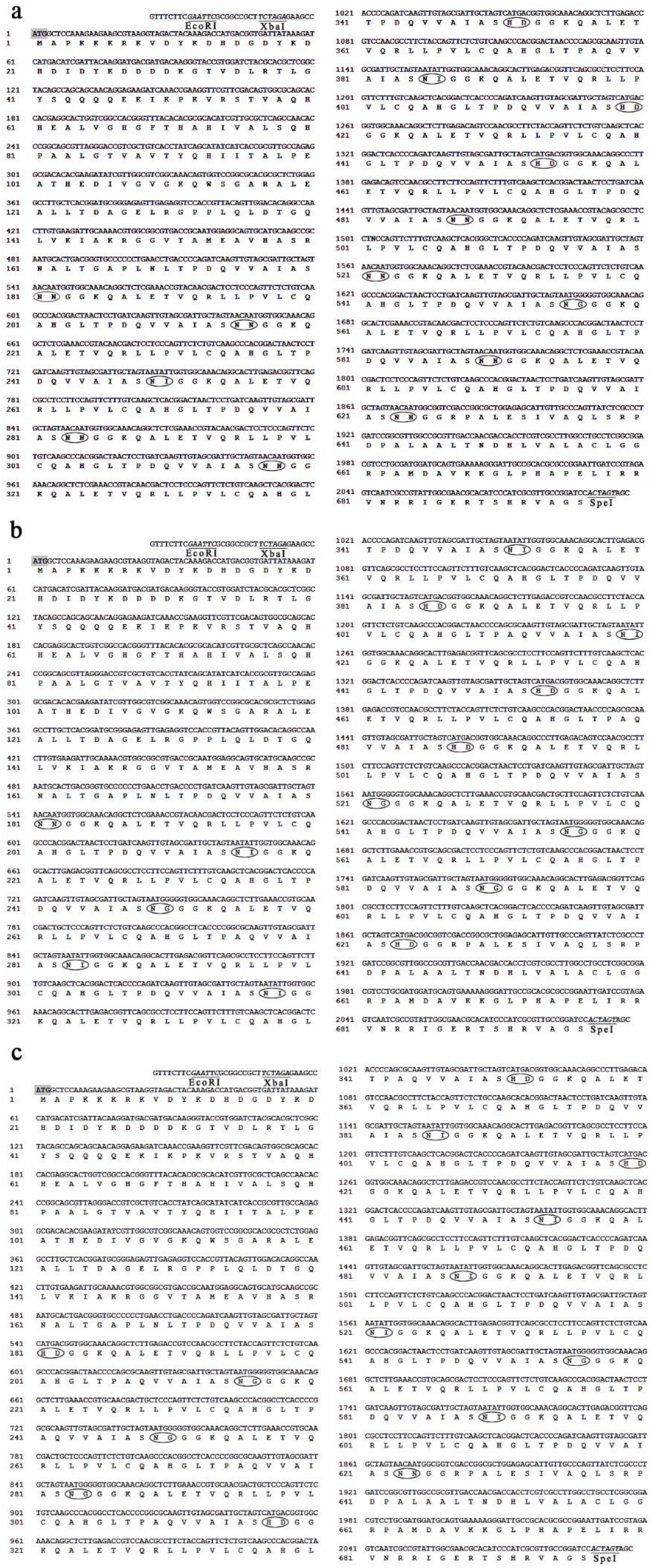
a
b
c
EcoRI
XbaI
SpeI

Supplementary Figure S4. Encoding sequence and deduced amino acids of TALE1 (**a**), TALE2 (**b**) and TALE3 (**c**). The start codon is shaded. The RVDs of TALE modules are circled. The restriction sites are marked in italic and underlined.

Supplementary Table S1. Primers used in the experiments

Primer name	Sequence (5'-3')	Application
GFP1-F	CGGAATTCGCGGCCGCTTCTAGAATGC	GFP1 gene expression
GFP1-R	GGACTAGTATTTCTCCTCTTTAATCTCTAG TATTATTGTTTGTCTG	GFP1 gene expression
GFP2-F	CGGAATTCGCGGCCGCTTCTAGAAAGAAT GGAATC	GFP2 gene expression
GFP2-R	GGACTAGTTTATTATTTGTATAGTTC	GFP2 gene expression
IAAM-F	GGAATTCGCGGCCGCTTCTAGAGATGTTT GGACCGG	IAAM gene expression
IAAM-R	GCGGCGGACTAGTCTTATTAGTCCCCCAG CG	IAAM gene expression
IAAH-F	GGAATTCGCGGCCGCTTCTAGAGATGCGC GAAATG	IAAH gene expression
IAAH-R	GCGGGCGGCGGACTAGTCTTATTAGCCTT TTAACAC	IAAH gene expression
RBS30-F	AATTCGCGGCCGCTTCTAGAGATTAAAGA GGAGAAATA	RBS30 annealing
RBS30-R	CTAGTATTTCTCCTCTTTAATCTCTAGAAG CGGCCGCG	RBS30 annealing
pSB1C3-F	GAACCTCTTACGTGCCCGATCAA	Colony PCR Detection
pSB1C3-R	CGCCGCAGCCGAACGAC	Colony PCR Detection

TALE-F	CTTGTGAAGATTGCAAAACGTGGC	TALE module identification
TALE-R	GGACGTCCGCCGAGGCAGGCCAAG	TALE module identification
ChIP-P1	ATGCGTAAAGGAGAAGA	ChIP assay
ChIP-P2	TTATTGTTTGTCTGCCA	ChIP assay
ChIP-P3	AAGAATGGAATCAAAGT	ChIP assay
ChIP-P4	TTATTATTTGTATAGTT	ChIP assay
GFP1-rtF	CTACTTTCGGTTATGGTGTT	Semi-qRT-PCR and qRT-PCR
GFP1-rtR	GTCTTGTAGTTCCCGTCATC	Semi-qRT-PCR and qRT-PCR
GFP2-rtF	AGCAGACCATTATCAACAAA	Semi-qRT-PCR and qRT-PCR
GFP2-rtR	TACAAACTCAAGAAGGACCA	Semi-qRT-PCR and qRT-PCR
IAAM-rtF	TTGAAAGTCGTCCGTTCCCTA	Semi-qRT-PCR and qRT-PCR
IAAM-rtR	CGAATGGCCTGTTGAATATCTC	Semi-qRT-PCR and qRT-PCR
IAAH-rtF	TGACTTAGATGGCGAAGTAGAACG	Semi-qRT-PCR and qRT-PCR
IAAH-rtR	CACCGTGAAGGTTAATGTCTGG	Semi-qRT-PCR and qRT-PCR
16s-27F	GAGAGTTTGATCCTGGCTCAG	Semi-qRT-PCR and qRT-PCR
16s-519R	GWATTACCGCGGCKGCTG	Semi-qRT-PCR and qRT-PCR
